# Genome-wide profiling of the human papillomavirus DNA integration in cervical intraepithelial neoplasia and normal cervical epithelium by HPV capture technology

**DOI:** 10.1038/srep35427

**Published:** 2016-10-19

**Authors:** Ying Liu, Chaoting Zhang, Weijiao Gao, Limin Wang, Yaqi Pan, Yunong Gao, Zheming Lu, Yang Ke

**Affiliations:** 1 Key Laboratory of Carcinogenesis and Translational Research (Ministry of Education/Beijing), Laboratory of Genetics, Peking University Cancer Hospital & Institute, No. 52 Fucheng Road, Haidian District, Beijing 100142, China; 2 Key laboratory of Carcinogenesis and Translational Research (Ministry of Education/Beijing), Department of Gynecologic Oncology, Peking University Cancer Hospital & Institute, No 52, Fucheng Road, Haidian District, Beijing 100142, China; 3 Key laboratory of Carcinogenesis and Translational Research (Ministry of Education/Beijing), Laboratory of clinical laboratory, Peking University Cancer Hospital & Institute, No 52, Fucheng Road, Haidian District, Beijing 100142, China; 4 Key Laboratory of Carcinogenesis and Translational Research (Ministry of Education/Beijing), Department of Biochemistry and Molecular Biology, Peking University Cancer Hospital & Institute, No. 52 Fucheng Road, Haidian District, Beijing 100142, China

## Abstract

HPV integration plays an important role in cervical carcinogenesis. HPV genotypes and the exact integration sites were investigated using HPV capture technology combined with next generation sequencing in 166 women. Three, one and six integration sites were verified in 7 HPV-positive ‘normal cervical epithelium’, 6 HPV-positive CIN2 and 15 HPV-positive CIN 3 samples, respectively. Of the 10 integrations, one and nine were involved with HPV33 and HPV16, respectively. Our study accurately evaluated HPV integration level in CINs and normal cervical tissues using high-throughput viral integration detection method providing basic evidence for HPV integration-driven cervical carcinogenesis.

Human papillomavirus (HPV) infection has been recognized as an important cause of cervical precancerous lesions or cancer, and yet is necessary but not sufficient for cervical carcinogenesis process^1^,^2^. Therefore, in addition to HPV infection, HPV integration could contribute to the cervical carcinogenesis process.

HPV integration could upregulate the expression of viral oncogenes E6 and E7 and eventually promotes host genomic instability, which could be a crucial event of cervical carcinogenesis process^3^,^4^. Additionally, the level of HPV integration was positively associated with cervical intraepithelial neoplasia (CIN) grades and was proposed as a marker for cervical disease progress^5^,^6^. Therefore, comprehensively and accurately identifying the sites and level of HPV integrations from normal cervical epithelium to CIN and cervical cancer is necessary to assess HPV-induced carcinogenesis process. However, cervical HPV integrations have only been comprehensively investigated in invasive cancer and limited data were available in CINs and normal cervical epithelium^7^,^8^. Moreover, most previous assays could not identify HPV integrations sensitively, which could lead to the underestimation of HPV integration level^7^,^9^,^10^. Recently, the study by Hu *et al*. reported 3,667 HPV integration events in 26 CINs, 104 cervical cancer samples, and five cell lines based on high-throughput viral integration detection (HIVID)^8^. However, Nigel Dyer *et al*. claimed that 87% of the integration breakpoints reported by Hu *et al*. were likely to be experimental and computational artifacts according to their own data analysis pipeline^11^. Meanwhile we used the same HPV capture technology and the next generation sequencing as in Hu’s study to detect cervical HPV integrations in 39 HPV-positive primary cervical tumor samples and 2 cell lines and yet only identified 117 unique validated HPV integration breakpoints^12^. Moreover, we found that the Sanger sequencing validation rate based on one, two, three, or more than three discordant paired-end reads was 3.7%, 47.8%, 44.4%, and 83.3%, respectively, indicating that HIVID could be a sensitive method to detect integrated HPV and yet has a high false positive rate with fewer supporting reads. HPV integration rates in the study by Hu *et al*. could be overestimated in cervical cancer and even in CINs. Therefore, given that comprehensive and accurate data regarding HPV integrations in CINs and normal cervical tissues were limited, we detected HPV integrations in a series of CINs and normal cervical epithelium samples using base-resolution HPV capture technology and the next generation sequencing as previously reported^12^.

In this study we enrolled 166 participants with CIN or ‘normal cervical epithelium’ in order to investigate the level of cervical HPV integration in CINs and normal cervical epithelium.

## Result

### Distribution of HPV types

**Figure 1:**
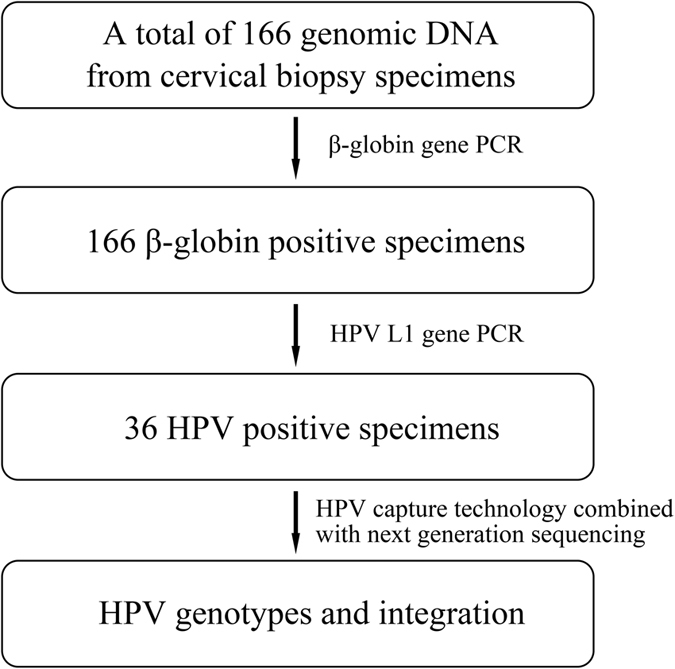
Flow chart of study design, sample selection and HPV detection.

**Figure 2:**
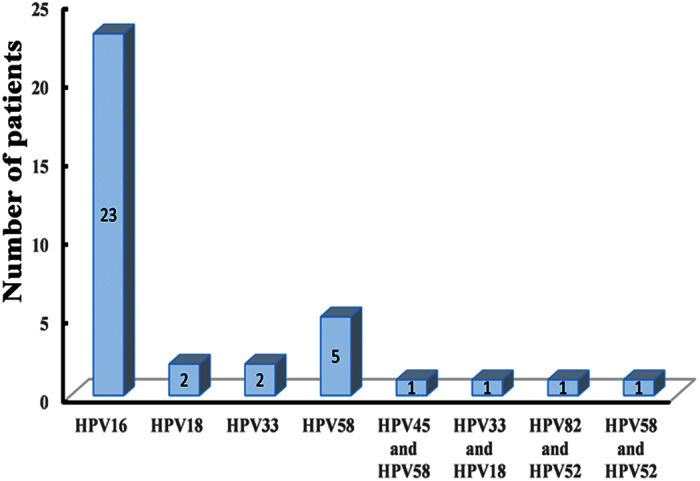
Distribution of HPV types among 36 HPV positive samples.

As shown in Fig. 1 and Table 1, of the 64 ‘normal cervical epithelium’, 62 CIN 1, 19 CIN 2 and 21 CIN 3 samples, we detected HPV in 7 (10.9%, 95% CI: 4.5–21.2%), 8 (12.9%, 95% CI: 5.7–23.9%), 6 (31.6%, 95% CI: 12.6–56.6%) and 15 (71.4%, 95% CI: 47.8–88.7%) samples, respectively. Of the 166 samples, we detected HPV16 (n = 23), HPV18 (n = 2), HPV33 (n = 2) and HPV58 (n = 5); four samples harbored two types of HPV with HPV45 and HPV58 in CIN 1–42, HPV 33 and HPV18 in CIN 1–62, HPV 82 and HPV52 in CIN 2–12, and HPV58 and HPV52 in CIN 3–5 (Fig. 2). HPV infection rate in younger women (age< = 50) and in older women (age > 50) were 25.2% and 12.8% (*P* = 0.096 by Fisher’s exact test).

### Determination of potential HPV integration sites

As described in our previous study^12^, if a specific site had one or more discordant reads mapped on one end to the HPV reference genome and the other to human chromosome, it would be considered as a potential HPV integration locus. A total of 37, 21, 44 and 45 potential HPV integrations were identified in 7 HPV-positive ‘normal cervical epithelium’, 8 HPV-positive CIN1, 6 HPV-positive CIN2 and 15 HPV-positive CIN 3 samples, respectively (Table 1).

### Validation of HPV integration sites

In order to confirm the potential HPV integration sites and to further identify the sequence between cellular and viral genome, all potential HPV integration positions were verified by targeted PCR amplification and Sanger sequencing. The validation rate of Sanger sequencing on the basis of one, two, three, or more than three different paired-end reads, was 0% (0/131), 33.3% (95% CI: 0.8–90.6%), 50% (95% CI: 1.3–98.7%), and 72.7% (95% CI: 39.0–94.0%), respectively (Table 1). After validation, only three, zero, one and six integration sites were verified in 7 HPV-positive ‘normal cervical epithelium’, 8 HPV-positive CIN1, 6 HPV-positive CIN2 and 15 HPV-positive CIN 3 samples, respectively. Of the 10 integrations, one was involved with HPV33 and nine with HPV16, respectively (Table 1).

### Mapping and characterization of cellular-viral junction sequences

**Figure 3:**
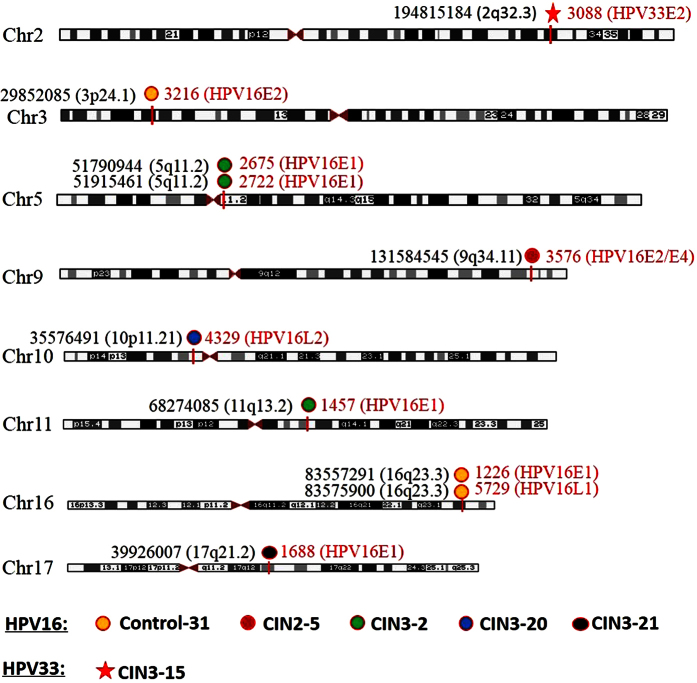
Chromosome localization of the 10 HPV integration breakpoints in six samples. The chromosomal reference of all viral-cellular breakpoints with respect to Giemsa-stained bands was taken from the UCSC databases. Six HPV-positive samples with integration breakpoints are shown in unique color. Cell sequence breakpoints are marked with black and HPV breakpoints are marked with red.

10 unique HPV integration breakpoints were distributed in six samples, including ‘normal cervical epithelium’ (n = 1), CIN2 (n = 1), and CIN3 (n = 4) (Fig. 3 and Table 1). Of the 10 integrations, four samples (CIN2-5, CIN3-15, CIN3-20, and CIN3-21) had one integration site and two samples (Control-31 and CIN3-2) had three integration sites, respectively (Fig. 3 and Table 1). The viral genome regions of the 10 validated integration positions in six samples were E1 (n = 5), E2 (n = 2), E2/E4 (n = 1), L1 (n = 1), and L2 (n = 1). Due to the limited number of integration events, we did not find hot spots in the human genome (Table 1).

All integration positions were examined for the presence of fragile sites in the human genome. Of the 10 integration positions, one was located in a fragile site and three were close to a fragile site (Supplementary Table S1). Meanwhile, the human genomic sequences within 50 kb of an integration locus were investigated. Seven integration sites were located in cellular genes with six in introns and one in an exon (Supplementary Table S1).

## Discussion

Our study only found three, zero, one and six verified integration sites in 7 HPV-positive ‘normal cervical epithelium’, 8 HPV-positive CIN1, 6 HPV-positive CIN2 and 15 HPV-positive CIN 3 samples, respectively. HPV integration sites were mainly located in the E1 and E2 regions of the viral genome and in cellular genes of the human genome.

We found that HPV integration rates in HPV-positive CIN 1, CIN 2, and CIN 3 samples were 0% (0/8), 16.6% (95% CI: 0.4–64.1%), and 26.7% (95% CI: 7.8–55.1%), respectively, which were similar to those reported previously^7^,^13^,^14^. However, the study by Hu *et al*.^8^ reported that HPV integration rates in CIN 1, CIN 2, and CIN 3 were 50% (95% CI: 18.7–81.3%), 44.4% (95% CI: 13.7–78.8%), and 71.4% (95% CI: 29.0–96.3%), respectively, which were much higher than the validated integration rates in our study and yet similar to what we considered as the “potential” HPV integration rates (37.5% [95% CI: 8.5–75.5%], 66.7% [95% CI: 22.3–95.7%], and 53.3% [95% CI: 26.6–78.7%]) based on HPV capture and the next generation sequencing technology without Sanger sequencing validation. Notably, the validation rate of Sanger sequencing on the basis of one, two, three, or more than three different paired-end reads was 0% (0/131), 33.3% (95% CI: 0.8–90.6%), 50% (95% CI: 1.3–98.7%), and 72.7% (95% CI: 39.0–94.0%) in our study, respectively. Moreover, HPV integration rates in cervical cancer was significantly higher in Hu’s study^8^ than in our previous study^12^. The overall HPV integration rates in the study by Hu *et al*. could be overestimated in cervical cancer and CINs, since only selected HPV integrations in Hu’s study were validated by Sanger sequencing.

It is worth noting that HPV integrations could occur in cervical tissue with normal epithelium and the integration rate in CIN 3 was significantly higher than those in CIN 1 or CIN 2. This indicated that HPV integrations could play an important role in the early stage of cervical carcinogenesis, although our results were lack of the statistical analysis and the sample size may not be enough for the analysis. In addition, we found similar characteristics of the HPV integration sites in cervical cancer and non-cancer specimens^12^. For example, HPV integration sites were mainly located in the E1 and E2 regions of the viral genome and in cellular genes of the human genome.

Two different types of mechanisms are presumed to explain cervical carcinogenesis process induced by HPV integration, i.e. altering viral gene expression or disrupting cellular transcripts. In order to determine the effect of these two mechanisms, it is necessary to comprehensively profile HPV integrations in host and viral genome. However, detection methods of HPV integration in most previous studies were low-throughput and lower sensitivity. In order to better understand the cervical carcinogenesis induced by HPV integration, this approach is able to discern fusion breakpoints accurately at single-base resolution for further elucidating the effect of HPV integration on viral and its flanking cellular transcripts. In addition, since HPV integration could lead to the viral persistence and moreover HPV persistent infection plays role in cervical carcinogenesis, this approach provides unbiased, genome-wide integration information to monitor the persistent or permanent infection.

However, there are some limitations in our study. Firstly, since HPV integration rates in ‘normal cervical epithelium’ and CIN were low and moreover only 36 HPV-positive women were involved in HPV integration analysis, comprehensively evaluating the sites and the level of HPV integrations was limited to some extent. Secondly, the cross-sectional study did not investigate the temporal relationship between HPV integration and CINs. Thirdly, in our study, CIN enriched tissue was not sampled by laser microdissection, which did not rule out contamination from normal adjacent epithelium or the underlying stroma. This would overestimate HPV integration rate in CINs to some extent although this effect was small due to significantly lower HPV integration rate in ‘normal cervical epithelium’ than in CINs. Fourthly, Since HPV DNA was detected using a highly sensitive PCR primer set (SPF1/GP6+) amplifying a 184-bp fragment of the L1 open-reading frame before performing HPV capture and sequencing, this might produce HPV false negatives from L1 breakpoints among the 166 samples. However, since proportion of breakpoints occurring in this targeted region of L1 was low^12^ and in most situations, HPV viral genome may be existed in both episomal and integrated forms, HPV false negative probability due to L1 breakpoints was small.

In summary, the accurate identification of HPV integrations in CINs and normal cervical tissues could provide basic evidence for HPV integration-driven cervical carcinogenesis and be served as individualized markers in cervical cancer screening in the future.

## Materials and Methods

### Study population and specimen collection

A total of 166 cervical biopsy specimens were collected and diagnosed with normal cervical epithelium or acute/chronic cervicitis without atypical hyperplasia (n = 64), CIN 1 (n = 62), CIN 2 (n = 19) and CIN 3 (n = 21) from Beijing Cancer Hospital, Beijing, China, between 2014 and 2015. All biopsy specimens were reviewed by two experienced pathologists who confirmed the diagnosis of CIN. Cervical biopsies were histologically diagnosed using criteria defined by the World Health Organization^15^. All cases have no histological evidence of epithelial malignancy of the cervix in this study. Normal cervical epithelium or acute/chronic cervicitis without atypical hyperplasia is defined as ‘normal cervical epithelium’ in this study. Punch biopsy samples were divided into two parts; one was kept for histopathological analysis, and the other one was used for HPV typing and integration analysis. Individual informed consents had been collected from all participants. This study received ethical approval from the Institutional Review Board of the Peking University School of Oncology, China. All experiments were performed in accordance with relevant guidelines and regulations.

The specimens were stored at −80 °C and genomic DNA was extracted from the frozen tissues using DNeasy Blood & Tissue Kit (Qiagen, Hilden, Germany) following the manufacturer’s instructions. The β-globin gene was evaluated in all specimens by PCR.

### HPV typing and integration detection

HPV DNA in valid (β-globin positive) specimens was detected using a highly sensitive PCR primer set (SPF1/GP6+) amplifying a 184-bp fragment of the L1 open-reading frame^16^. Specimens showing the PCR amplification product were used to identify HPV genotypes and integrations. HPV probes were designed according to the full-length genome of 17 HPV types (6, 11, 16, 18, 31, 33, 35, 39, 45, 52, 56, 58, 59, 66, 68, 69, and 82) by MyGenostics (MyGenostics, Baltimore, MD, USA). Details of HPV typing and the detection of HPV integrations, as well as Sanger sequencing validation of potential HPV integration sites were described previously^12^. In brief, the whole-genomic libraries were hybridized with HPV probes (MyGenostics GenCap Technology), adsorbed onto the beads via biotin and streptavidin magnetic beads, and the uncaptured DNA fragments were removed by washing. Then the eluted fragments containing the targeted gene were enriched by PCR to generate libraries for sequencing. Libraries were quantified and sequenced for paired-end 125 bp using the Illumina HiSeq 2500 sequencer (Illumina Inc., San Diego, CA, USA). Illumina clean reads were mapped to human genome (GRCh37/hg19) and HPV genome of 17 types using the BWA program. The paired-end read, uniquely mapped with one end to a human chromosome and the other to the HPV reference genome, is identified as a discordant read pair. If a specific position has one or more discordant read pairs, it would be considered as a potential HPV integration site. PCR and Sanger sequencing were used to verify all the potential HPV integration breakpoints. All sequences of the fusion genes were characterized by the NCBI human mega Blast database alignment tool and the UCSC Blat database.

### Statistical analyses

Fisher’s exact test was used to determine the relationship between age and HPV infection. Statistical analyses were performed using STATA version 12.0 software (STATA Corporation, College Station, TX, USA). *P* values less than 0.05 (two-sided) were considered to be significant.

## Additional Information

**How to cite this article**: Liu, Y. *et al*. Genome-wide profiling of the human papillomavirus DNA integration in cervical intraepithelial neoplasia and normal cervical epithelium by HPV capture technology. *Sci. Rep.* **6**, 35427; doi: 10.1038/srep35427 (2016).

## Supplementary Material

Supplementary Information

## Figures and Tables

**Table 1:** Summary of 36 HPV positive samples analyzed in this study: HPV integration status and clinical information.

Sample	CIN status	HPV type	Sequencing depth	No. of validated integration sites on the basis of different reads					HPV breakpoint (ORF)^a^	Cellular sequence breakpoint (map)^b^
				Total	1	2	3	≥4		
Control-6	Normal epithelium	16	13553							
Control-27	Normal epithelium	18	20434	0/28	0/28					
Control-30	Normal epithelium	16	5981							
Control-31	Normal epithelium	16	10695	3/5	0/2			3/3	1226 (E1) 3216 (E2) 5729 (L1)	83557291 (16q23.3) 29852085 (3p24.1) 83575900 (16q23.3)
Control-36	Normal epithelium	16	2638							
Control-54	Normal epithelium	18	5830	0/4	0/4					
Control-59	Normal epithelium	58	7289							
CIN1-16	CIN1	16	4917							
CIN1-19	CIN1	58	4061	0/14	0/14					
CIN1-34	CIN1	16	15714	0/5	0/5					
CIN1-42^c^	CIN1	45 and 58	615 and 93							
CIN1-43	CIN1	58	3879	0/2	0/2					
CIN1-55	CIN1	16	7111							
CIN1-61	CIN1	16	3834							
CIN1-62^d^	CIN1	33 and 18	607 and 219							
CIN2-4	CIN2	58	7358	0/38	0/38					
CIN2-5	CIN2	16	2935	1/1				1/1	3576 (E2/E4)	131584545 (9q34.11)
CIN2-11	CIN2	16	8132	0/1	0/1					
CIN2-12^e^	CIN2	82 and 52	51384 and 12973	0/4	0/4					
CIN2-16	CIN2	16	4326							
CIN2-19	CIN2	33	7214							
CIN3-2	CIN3	16	3663	3/5	0/1	1/1	1/1	1/2	2675 (E1) 2722 (E1) 1457 (E1)	51790944 (5q11.2) 51915461 (5q11.2) 68274085 (11q13.2)
CIN3-3	CIN3	16	6061							
CIN3-4	CIN3	16	12228							
CIN3-5^f^	CIN3	58 and 52	11902 and 2613	0/25	0/24	0/1				
CIN3-6	CIN3	16	4010							
CIN3-7	CIN3	16	3582	0/2		0/1	0/1			
CIN3-8	CIN3	16	5572							
CIN3-10	CIN3	16	1014							
CIN3-12	CIN3	16	12659	0/1	0/1					
CIN3-14	CIN3	16	4306							
CIN3-15	CIN3	33	2126	1/2				1/2	3088 (E2)	194815184 (2q32.3)
CIN3-16	CIN3	16	8805	0/1	0/1					
CIN3-17	CIN3	58	1037							
CIN3-20	CIN3	16	12642	1/8	0/6			1/2	4329 (L2)	35576491 (10p11.21)
CIN3-21	CIN3	16	2211	1/1				1/1	1688 (E1)	39926007 (17q21.2)

Abbreviations: CIN, cervical intraepithelial neoplasia; HPV, human papillomavirus; ORF, Open Reading Frame.

^a^Nucleotide position of viral-cellular breakpoints on the HPV genome (Alignment to NC_001526.2 for HPV16 and M12732.1 for HPV33).

^b^Cellular nucleotide position of viral-cellular breakpoints on the human genome (Alignment to Hg19 human reference genome).

^c^The sample of CIN1-42 harbors HPV45 and HPV58, and the sequencing depth of HPV45 is 7-fold greater than that of HPV58. So, only HPV45, a main type of HPV in sample of CIN1-42, is analyzed in subsequent HPV assay.

^d^The sample of CIN1-62 harbors HPV33 and HPV18, and the sequencing depth of HPV33 is 2-fold greater than that of HPV18. So, only HPV33, a main type of HPV in sample of CIN1-62, is analyzed in subsequent HPV assay.

^e^The sample of CIN2-12 harbors HPV82 and HPV52, and the sequencing depth of HPV51 is 4-fold greater than that of HPV52. So, only HPV51, a main type of HPV in sample of CIN2-12, is analyzed in subsequent HPV assay.

^f^The sample of CIN3-5 harbors HPV58 and HPV52, and the sequencing depth of HPV58 is 4-fold greater than that of HPV52. So, only HPV58, a main type of HPV in sample of CIN3-5, is analyzed in subsequent HPV assay.
