# Supplementary material for: Genome-wide profiling of the human papillomavirus DNA integration in cervical intraepithelial neoplasia and normal cervical epithelium by HPV capture technology
Source: Sci Rep. 2016 Oct 19;6:35427. doi: 10.1038/srep35427 (PMC5069689; doi:10.1038/srep35427)
Supplement: Supplementary Information [file srep35427-s1.doc]

**Title:**

**Genome-wide profiling of the human papillomavirus DNA integration in cervical intraepithelial neoplasia and normal cervical epithelium by HPV capture technology**

Ying Liu^1†^; Chaoting Zhang^1†^; Weijiao Gao^2^; Limin Wang^3^; Yaqi Pan^1^; Yunong Gao^2^; Zheming Lu^4†*^; Yang Ke^1*^

| Supplementary Table S1. HPV-cellular DNA junctions confirmed by PCR amplification and Sanger sequencing | | | | | | |
| --- | --- | --- | --- | --- | --- | --- |
| Sample | Fragile site | class^a^ | name | family | Neighboring genes^b^ | Junction sequence^c^ |
| Control-31 | FRA16D (16q23.2) 1.9Mb distance | No |  |  | CDH13,inside=intron8 (AS) | GCAACCGGATATGCCCACTGAGGTGGAATCTGAGCTCTACTTTGTTTTTCTATACATATAGCTTTTAATCTAGG |
| Control-31 | FRA3A (3p24.2) 3.5Mb distance | No |  |  | RBMS3,inside=intron6 (AS) | AAATGAAAAGGAAAATGCCAAAGGATAGTCAACTTGACCCTCTACCACAGTTACTGATGCTT |
| Control-31 | FRA16D (16q23.2) 1.9Mb distance | DNA | MER5B | hAT-Charlie | CDH13,inside=intron8 (AS) | AATTCCTATCAGAATCTGGTTTTGTTTTTGTTTAAGGCTTCCAAACATATATTATCATGCAGGAACATCCAGACTACT |
| CIN2-5 | - | LINE | L2b | L2 | C9orf114,inside=exon12 (SE) | AGGACAGATCAGGGAAGGCTGCCTGACACAAAGGACGGATTAACTGTAATAGTAACACTA |
| CIN3-2 | - | LTR | LRT16A1 | ERVL | No gene within 50kb | GGAGAAAATAACAGAAGCAAACAGGGAAGCTCATACACTGGATTTCCGTTTTCGTCAAATGG |
| CIN3-2 | - | No |  |  | No gene within 50kb | AGCTAGAGACTGTTCATGTCAGAGATCAGGAATCTGGACCACGTCCTTGAGAAAAAGG |
| CIN3-2 | FRA11H (11q13) | LINE | L1MA7 | L1 | PPP6R3,inside=intron1 (AS) | TTGGAAATTCTCTTTGTGATTTTGAAATGCTAGTACATTTAAAATATTTGTAAGTGGTGTTT |
| CIN3-15 | - | No |  |  | No gene within 50kb | TTTTGAAATGTTAATCCAAGAGTCATACTCTTGTTTTTTAAAACATTTTGGTGGTTCACAAA |
| CIN3-20 | - | LINE | L1ME1 | L1 | CCNY,inside=intron3 (AS) | TTTTCTTTCCGTATTAATTTTGCCTGTTCTAGAATTATACCTAAGGTTGAAGGCAAAACTATTGCTGATCA |
| CIN3-21 | - | No |  |  | JUP, inside=intron2 (SE) | ACTGGGGAGCAGGGCAGGCCACAGACAGTCCACAACCATTCCCCATGAACATGCTAAACTTTG |
| ^a^ The class of repeating elements. LINE, long interspersed nuclear elements; SINE, short interspersed elements; LTR, long terminal repeat elements. | | | | | | |
| ^b^ The neighboring genes located within 50kb of the viral-cellular junction. | | | | | | |

^c^ Red letters, HPV sequence (Alignment to NC_001526.2 for HPV16, NC_001357.1 for HPV18, and D90400.1 for HPV58); blue, Nucleotides that did not align to HPV or cellular sequence; black, Cellular sequence (Alignment to Hg19 human reference genome); green, Nucleotides shared between virus and host genomes.
